# Expanding the Romantic Circle

**DOI:** 10.1007/s10677-020-10114-y

**Published:** 2020-08-15

**Authors:** Tena Thau

**Affiliations:** Faculty of Philosophy, University of Oxford, Woodstock Road, Oxford, OX2 6GG UK

**Keywords:** Appearance-based discrimination, Attraction, Enhancement, Queer rights

## Abstract

Our romantic lives are influenced, to a large extent, by our perceptions of physical attractiveness – and the societal beauty standards that shape them. But what if we could free our desires from this fixation on looks? Science fiction writer Ted Chiang has explored this possibility in a fascinating short story – and scientific developments might, in the future, move it beyond the realm of fiction. In this paper, I lay out the prudential case for using “attraction-expanding technology,” and then consider it from a moral point of view. Using the technology would, in one respect, be morally good: it would benefit those whom prevailing beauty standards marginalize. But attraction-expanding technology also raises a moral concern – one that can be cast in non-harm-based and harm-based terms. I argue that the non-harm-based objection should be rejected, because it is incompatible with a moral principle central to queer rights. And the harm-based objection, I argue, is outweighed by the benefits of attraction-expanding technology, and undermined by the prerogative you have over your personal romantic choices. I conclude by considering whether, from the perspective of society, the development of attraction-expanding technology would be desirable.

If you experience romantic^1^ attraction, physical appearance probably influences who you feel it *for*. For example, you’re likely more attracted to people who have a face that is highly symmetrical, and close to the population mean (Fink and Penton-Voak 2002). You might have preferences regarding the height of your potential partners. And perhaps you only feel attraction for people with a masculine, or feminine, or androgynous look.

As a consequence, of the population of people who are looking for intimacy or love, capable of consenting to it with you, and compatible with you personality-wise, there may be a significant fraction that you would not even consider, if you were looking for a partner. If you saw them on Tinder, you would swipe left.

But what if there was a drug you could take that would broaden your romantic ‘type’?

In this paper, I will explore whether it would be ethical, and prudentially wise, to use future biomedical technology to reduce, or eliminate, the influence of physical appearance on one’s romantic desires. I will also consider whether the development of such a technology would be beneficial, from the perspective of society.

In Part I, I introduce attraction-expanding technology (AET), and explain how it differs from attraction-altering technologies that philosophers have imagined before. In Part II, I lay out the prudential case for taking AET, and in Part III, I turn to ethical considerations. I first identify a moral benefit: taking AET would reduce the romantic and societal discrimination faced by those who are marginalized by prevailing standards of beauty. I then raise a moral concern – one that can be cast in non-harm based and harm-based terms. I argue that the non-harm-based objection should be rejected, because it is incompatible with a moral principle central to queer^2^ rights. On the harm-based objection, I offer two lines of response. First, I argue that the benefits of AET overwhelm its potential harms. Second, I argue that individuals have a strong moral prerogative over their personal romantic choices. In Part IV, I consider whether, from a societal perspective, the development of AET would be desirable, and speculate on the radical changes that could come about with widespread use.

## Background

In his thought-provoking short story “Liking What You See,” Ted Chiang (2014) imagines a neurological device that renders users unable to discern differences in physical attractiveness. In Chiang’s world, people can still feel sexual attraction, it is just a response to qualities deeper than looks.

The technological manipulation of romantic attraction might not be confined to science fiction for much longer. As Earp and Vierra (2018) write, “Based on current trends in research, it is not implausible that in the not-too-distant future, scientists will know enough about the genetic, epigenetic, neurochemical and other brain-level factors that are involved in shaping [attraction] that [it] could be effectively and relatively safely modified… with the application of some biotechnology.” And philosophers have begun to anticipate the ethical implications of such technology. Their focus has been, primarily, on its use within the context of an existing romantic relationship – to revive a fading one,^3^ or end an abusive one.^4^

But recent work has also explored the use of technology to reconfigure one’s attractions on a more basic level – changing the shape of one’s dating pool.^5^ Delmas and Aas (2018) argue that (in an ideally just society^6^) “the ability to expand or narrow, and experiment with, the range of one’s basic sexual preferences would enhance freedom of choice and autonomy for everyone.” In this paper, I will focus on technological interventions that would *expand* one’s romantic attractions. One way of doing this could be along the dimension of personality: by expanding the range of *personality profiles* that one finds attractive (or non-off-putting). But my focus will be on interventions that would expand one’s attractions along the dimension of *physical appearance*. In contrast to Delmas & Aas, whose case for attraction-*changing* technology is based on a general appeal to liberty, my case for attraction-*expanding* technology is grounded in individuals’ prudential interests, as well as in the impartial, moral reasons that we have to fight appearance-based discrimination.

Previous work in philosophy has advocated ‘low-tech’ ways of expanding attraction, for reasons grounded in social justice. Eaton (2016) proposes that people seek to cultivate an aesthetic appreciation for fat bodies through the viewing of fat-positive art – and contends that expanding “our collective taste in bodies” is critical to the fight against fat oppression. And Srinivasan (2018) argues that there may be “a duty to transfigure [our desires] as best we can” – to the extent that our desires are influenced by beauty standards rooted in racism, sexism, transphobia, and other prejudices. As Srinivasan writes, “our sexual preferences can and do alter, sometimes under the operation of our own wills – not automatically, but not impossibly either.”

But the battle against oppression, and oppressive beauty standards, need not be fought with our *wills* alone. In *The Xenofeminist Manifesto*, Laboria Cuboniks (2018) calls on feminists to deploy technology in the service of progressive political ends, declaring: “If nature is unjust, change nature!” I will consider the high-tech expansion of attraction in a similar spirit. AET could be a powerful force for social justice, as I will explore in Parts III and IV. But its value, too, lies in the benefits it would bring to individual users – their desires now freed from the influence of looks.

## The Prudential Case for Attraction-Expanding Technology

If you are looking for a romantic partner, or partners – for casual sex, love, or anything in between – then the prudential case for taking AET would be strong.

### Less Loneliness

Romantic loneliness can have a heavy toll on happiness and health.^7^ AET could be a medical cure: Since you would no longer be ruling out potential romantic partners on the basis of arbitrary physical characteristics, you would have a larger dating pool, and less difficulty finding a match. So you would be less likely to be single, or sexless, if you didn’t want to be.

### More Compatibility

It would be helpful to contrast AET with a different type of future technology: “love drugs.” Unlike AET, which would expand your dating pool, love drugs – as they are standardly understood in the literature – would be taken within the context of a relationship, to enhance partners’ feelings for each other. Proponents of love drugs have argued that taking them would be morally analogous to (and could be used alongside) widely-accepted non-biomedical methods of improving relationships, like couple’s therapy (Savulescu and Sandberg 2008; Earp et al. 2012; Earp 2019).

But love drugs would strike many as an unsettling prospect. A central concern – and one that the early proponents of love drugs anticipated (Savulescu and Sandberg 2008; Earp et al. 2012) – is that these drugs might be taken by partners who aren’t right for each other – conjuring up feelings of attachment between people who would be better off apart.

Thus, Sandberg and Savulescu acknowledge that, if people are not compatible with each other, using love drugs would be harmful, “merely serv[ing] to perpetuate a bad relationship” (p. 39).

But they maintain that love drugs could be beneficial in a wide range of cases – not only in the context of an existing, loving relationship (to help maintain the romantic ‘spark’), but even at a relationship’s inception, to “instil a new love” (p. 40) – provided that new lovers are not “fundamentally incompatible” (Earp et al.’s term, p. 568).

*“Not fundamentally incompatible”* might strike you as uncomfortably low bar. Many people hope to find love with someone who is an *exceptionally wonderful fit* for them. And even in casual encounters, people often want high compatibility; electric conversational chemistry and a shared sense of humour can enhance the experience of a one-night stand, or short-term fling.

And even if we would be willing to partner, or hook-up with, someone who barely makes it over the low bar of “not fundamentally incompatible,” we would probably prefer it if, ceteris paribus, they soared above it.^8^

While the importance of personality-compatibility gives you a reason to be wary of taking *love drugs* (though not necessarily a decisive one), it *supports* the case for taking *AET*. By opening yourself up to dating people of all appearances, you would be able to optimize, in your romantic search, for personality-fit.

You might choose not to optimize for personality-fit, however. Perhaps you specifically seek partner that you are not too compatible with – for instance, to avoid the risk of heartbreak. And you might care about characteristics of your partner, in addition to their personality (though this partially depends on how broadly you construe the word). For example, you might seek a partner who would be a good parent, or who could provide you with financial security. But whatever your preferences, the prudential case for taking AET still applies: It would be easier to find a partner who is at your ideal level of compatibility – wherever it is that you set it – and who embodies all of the qualities you are looking for – whatever those qualities are – if you are not ruling out potential partners on the basis of their looks.

Perhaps there is even a person you already know, who has expressed interest in you, and who you think you would be very compatible with – if only they were, in some superficial (appearance-based) way, more your “type.”^9^

### A Prudential Concern

The objection might be raised that having more romantic options would make your life go worse, not better. With so many potential dates, you might be more likely to end a relationship at the first sign of any conflict, rather than putting in effort to try to get things back on track. And you might be less likely to commit to a relationship in the first place, if you were on AET. Why settle down when you can enjoy playing the (now vastly larger) field?^10^

I have three responses to this concern. The first is to challenge its “amatonormative”^11^ presuppositions. If, after taking AET, you decide to exit a relationship that is rocky, or choose to pass up an opportunity to begin a relationship, then our presumption – in the absence of any argument to the contrary – should be that you have made the decision that is best for you. Prolonging the romantic relationship that one is in, or seizing the first opportunity to start a relationship if one is single, are not universal and sacrosanct goals. Choosing to forgo a romantic relationship(s) in favour of causal dating, hooking up, or just enjoying one’s own company, are all valid choices that are compatible with having a happy and fulfilling life.

Second, *even if* relationship-duration is something that you want to maximize, it is possible that AET would lead to more and longer relationships, rather than fewer and shorter ones. As I explained in the previous section, AET would tend to increase romantic *compatibility* – and you might be eager to commit to your super-compatible partner.

Third, if – despite the previous two points – you still think that having more romantic options would worsen your life, it might nevertheless improve your life to *take AET*, provided that you also took steps to ensure that your dating pool did not increase in size. For example, you could reduce the radius of your romantic search; if you previously travelled up to 50 miles away to date, you could take AET, and find that you now have the same number of attractive prospective dates – now all within walking distance. Or, if you have experienced discrimination or discomfort on dating apps, but felt it necessary to use them anyway in order to meet potential partners, AET would make it easier for you to meet, offline, people that you are attracted to. In other words, if you want to keep your romantic options limited, it seems better to limit them in non-arbitrary ways that would enhance your life, rather than through the arbitrary filter of physical taste.

## The Moral Status of Attraction-Expanding Technology

### A Moral Benefit

By taking AET, you would benefit those who are marginalized by societal beauty standards. In much of the world, beauty standards that favour whiteness, thinness, and gender-normativity prevail. AET would directly reduce the *romantic* discrimination felt by those whose bodies do not fit these narrow and oppressive standards of beauty.

AET would reduce *non*-romantic, appearance-based discrimination as well. People judge those they perceive as less attractive to be less competent (Dion et al. 1972), less intelligent (Talamas et al. 2016), and less trustworthy (Shinners 2009) than their peers. And these biases exact a heavy financial toll; someone judged to be in the bottom one-seventh in attractiveness will, over the course of their lifetime, earn hundreds of thousands of dollars less than someone in the top third (Hamermesh 2011). But if AET use became widespread, then appearance-based discrimination would recede.

Eaton (2016) and Chiang (2014) both emphasize the difficulty of countering ‘lookism’ through education alone, and the importance of expanding people’s perceptions of attractiveness. Drawing on Aristotle, who believed that teaching moral principles should be supplemented with instilling the right moral *emotions*, Eaton argues that the fight against fat oppression should involve not only correcting stereotypes and misinformation (the health risks of being “overweight” are overstated by the media^12^,^13^), but also overcoming “our collective repulsion and disgust at fat.” Similarly, Chiang (through the character Joseph Weingartner) writes that, though “[the AET device] by itself can’t eliminate appearance-based discrimination [,] what it does, in a sense, is even up the odds; it takes away the innate predisposition, the tendency for such discrimination to arise in the first place. That way, if you want to teach people to ignore appearances, you won’t be facing an uphill battle.”

Since AET would help counter looks-based discrimination, you would not only have a prudential reason to take it, but a moral reason as well.^14^

### Moral Concerns

If the only effect of AET was to make you less picky about physical attractiveness, then it would not seem to raise any moral concern. Surely it would be morally *permissible* to make yourself less ‘superficial’ when it comes to who you date.

But a technology that made people less concerned, or not concerned at all, with the physical appearance of their potential partners could have a further effect in a subset of users – those users who are not already pansexual^15^: it might expand their sexual orientation.

When it comes to technologies that might, in the future, alter sexual orientation, the most salient danger is that they could be used as a tool of oppression. Historically, and to this day, queer people have been pressured and, in many cases, coerced, into undergoing “conversion therapies” – which are ineffective^16^ and can cause severe harm.^17^ But if *effective* orientation-changing technology is developed in the future, then it might reduce the size of queer populations – at worst, reversing the global trend (Felter and Renwick 2019) towards increased acceptance of queer rights, and resulting in more isolation and oppression for those who remained queer (Behrmann and Ravitsky 2014).

This is indeed a serious worry for any technology that would eliminate same-gender^18^,^19^ desire. But the specific technology that I am considering – *AET –* would not have that effect. AET, as I have defined it, would expand the set of people that you are attracted to, without eliminating any same-gender attractions that you already have.

It might be worried, though, that AET could be used in a similar way as same-gender-desire-eliminating technology. Some previously-gay people who took AET, and then became attracted to people of all genders, might, as a result of homophobic discrimination or internalized homophobia, choose to forgo same-gender relationships in favour of heteronormative relationships.^20^

Of course, even under the status quo, some gay people pursue heterosexual relationships for these reasons. (For them, the only change that AET would make would be to increase the satisfaction that they would derive from these relationships.) But it might be worried that some gay people, on the margin, would, after taking AET, retreat into heterosexuality, when they otherwise would have come to embrace their queerness.

Still, it does not seem *morally wrong* for someone to use AET in this way – especially if the discrimination that they would otherwise face would be acute. It is not morally incumbent, on individuals, to expose themselves to severe discrimination on the altar of social progress. In this vein, some queer writers and activists have challenged the mainstream narrative of “coming out,” and affirmed that the decision to remain closeted is a valid one. Vaid-Menon (2018) emphasizes the particular dangers that coming out can pose for queer people of color, and writes that those who “cannot [or] are not interested in ‘coming out’ [should not be] shamed and dismissed.”^21^

So, while some previously-gay users of AET might choose to seek refuge from a violently homophobic world in the safe-haven of heteronormativity, it would not be *morally wrong* for them to do so.

A different concern has to do with the use of AET by straight people. It might be thought that heterosexuals who took AET, and whose sexual orientation broadened as a consequence, would be wronging the queer community.^22^ This worry is motivated by the objections that are often raised to non-queer people entering queer spaces, like gay bars (e.g. Farber 2017). It can be cast in non-harm-based or harm-based terms.

#### The Non-Harm-Based Objection

According to the non-harm-based version of the worry, it would be *intrinsically* morally wrong for straight people to use AET– apart from any harms that might result, and even if no harms resulted at all. This might be because it is inherently wrong to ‘commodify’ queerness or ‘appropriate’ queer identity. (An appropriation-based objection, though, would stand in tension with elements of queer theory, which emphasize the instability and even incoherence of identity categories, and eschew their policing; see e.g. Butler 1990.)

But what is ironic about these non-harm-based objections is that – though they come from a place of concern for the queer community^23^ – they share a common thread with the queer community’s *enemies*: an insistence that actions that harm no one can nevertheless be morally wrong.

Opponents of queer rights will sometimes make appeals to alleged harms,^24^ but very often, their opposition to queer rights is grounded in the belief that queer identities and lifestyles are inherently immoral.

But supporters of queer rights can (and often do) offer the following compelling response: Queer people are not harming anyone by being queer, and actions that do not harm anyone cannot be morally wrong.^25^

Though the second part of this response – that harmless actions cannot be morally wrong – is not without controversy in the philosophical literature,^26^ it should be noted that one need not be a consequentialist to endorse it,^27^ and a wide-construal of harm^28^,^29^ can increase its appeal.^30^ And detractors of the principle face the lingering worry that their alleged “harmless wrongs” are simply bigotry and intuitive discomfort in philosophical dress^31^; and indeed, philosophers used to cite homosexuality as an example of a harmless wrong (see e.g. Feinberg 1990, p. 21).

This “absence of harm” response will become increasingly crucial to the case for queer equality if the advent of attraction-altering technology renders the popular moral justification – *“It’s not a choice”* – obsolete.^32^*“It’s not a choice”* is, in any case, an unnecessarily weak justification; it is wrong to discriminate even on the basis of those characteristics that we can choose, like our religious identification. Moreover, “*It’s not a choice”* (and its variant, *“Born this way”*) erase the lived experiences of those queer people who emphasize having agency over their orientation (like “political lesbians”; Leeds Revolutionary Feminist Group 1981) or who experience their orientation as changing over time (Diamond 2008).^33^ (The unaffirming *tone* of this justification has been criticized too; as Cuboniks 2018, powerfully writes, “[T]oo often we are told to seek solace in unfreedom, staking claims on being ‘born’ this way, as if offering an excuse with nature’s blessing…The time has now come to tear down [Nature’s] shrine…not bow down before it in a piteous apology for what little autonomy has been won.”)

To oppose orientation-expanding technology on non-harm-based grounds, therefore, would be to forfeit what may be the strongest moral defence of queer rights: that an act cannot be wrong – and personal liberty should not be infringed – if no one is being harmed.

#### The Harm-Based Objection

A different objection to the use of AET by heterosexuals would appeal to the ways in which it might *harm* the queer community. But how might the use of AET by straight people, be harmful to queer people?

If AET had short-lasting effects, then it is not difficult to imagine how it could be used in harmful ways. Non-queer people might expand their sexual orientation for a night, for fun, and then – out of ignorance, insensitivity, or ill-will – do or say things that were hurtful to queer people. Any queer person who has been to a gay bar has likely had troubling encounters with non-queer people – who make homophobic comments, laugh at people who express their gender in non-normative ways, or express amusement or shock if they are flirted with by someone of the same gender *in a gay bar* (see e.g. Orne 2017). I take it as a given that the use of AET in these highly insensitive or malicious ways would be morally wrong.^34^

(This is not to say that all efforts to temporarily expand one’s attractions would be necessarily harmful, or that the desire for casual intimacy and sexual exploration – even just for a night – is necessarily a frivolous one. To the contrary, casual kissing and hooking-up can have great value for those involved.^35^)

In the remainder of this section, I will focus my attention on the “good faith” use of AET – which assumes not only the absence of any bad intentions (e.g. to mock queer people), but also the presence of good intentions (to be respectful). However, even with this restricted focus on good faith use, a harm-based objection could still be raised.

Since “newly queer” AET users would lack the sort of experiences and struggles that most “naturally queer” people have gone through,^36^ they might be less able to understand, and more likely to be dismissive of, others’ experiences. For example, suppose that, at a queer meetup, someone talks about homophobic remark that was recently made to them, that they felt hurt by. A newly queer person in attendance might be more likely to dismiss, vocally, this person’s experience as minor. After all, a single “microaggression”^37^ can seem trivial, when it is considered in isolation – by someone who does not understand what it is like to endure them day after day.

So AET use could, indirectly, lead to some harms, even when users have no ill intent. But if these kinds of insensitive comments were the only harms that resulted from good-faith AET use, they would not be sufficient to render it morally wrong. This is because these harms are overwhelmed in significance by the benefits that AET would unlock: By reducing lookism in society, AET would improve the lives, in virtually every dimension, of those who are marginalized by society’s beauty standards – while striking a blow at racism, sexism, and transphobia in the process. And for the individual user, AET could be life-changing, helping them have wonderful romantic experiences with people who they would have otherwise overlooked.^38^

Furthermore, a common moral view is that individuals are morally permitted to give special weight to their own interests (Scheffler 1992) – at least for those elements of their life that they value most deeply, like their love life. When it comes to individuals’ personal romantic choices, we normally do not think that negative side effects on others – for instance, the sadness your partner would feel if you break up with them – are sufficient to render those decisions morally wrong.

But a closer analogy will drive home the point. Suppose that a person has previously only had heterosexual romantic experiences, but they recognize that they are also capable of feeling same-gender attraction. Surely it would not be morally wrong for that person to decide to date or experience consensual casual intimacy with someone of the same gender, for the first time. Yet the very same harm-based objection that was raised against AET could be raised in this case; someone who has previously only had heterosexual experiences, and has had no previous involvement in the queer community, would be less sensitive to queer issues, and more likely to inadvertently cause offense.

What this example makes clear is that individuals have a strong moral prerogative over how they choose to live their own romantic lives.^39^ The choice to use AET, like the choice to date someone of the same gender for the first time, is not rendered morally off-limits by the non-zero risk of inadvertently causing offense. Provided that a person is acting in good faith, using attraction-expanding technology is their moral right.

## Attraction-Expanding Technology from a Societal Perspective

From the perspective of the individual, I have argued that it would be prudentially desirable and morally permissible to take AET, if it becomes available in the future.

But should AET be developed at all? From “the point of view of the universe” – to borrow Sidgwick’s (1874) phrase – would the development and widespread adoption of AET be good?

Some of the arguments that I have already made support an affirmative answer. We should expect to see loneliness decline, and happy romantic relationships flourish, in a world where AET is widespread. Hedonic adaptation^40^ thwarts many of our efforts to improve our lives – but given the clear relationship between romantic loneliness and unhappiness^41^ – AET could be a real lever through which lasting, population-wide increases in well-being could be achieved.

And the argument about lookism applies here too: reducing appearance-based discrimination is not only a moral reason for individuals to take AET, but a reason why society, as a whole, stands to benefit from its introduction.

But these are far from the only effects that AET would have on society. Given AET’s capacity to affect sexual orientation, we should be especially mindful of what its development would mean for the queer community. I touched on some of these effects in the last section, but here I will consider some of the more radical changes that might come about, if very large numbers of people took AET.

### A Post-Sexual Orientation World?

One possibility is that the queer community might fade into the mainstream, and sexual-orientation would cease to be a socially salient marker of identity.

The queer community today is not merely a group of people who happen to have non-normative sexual orientations, genders, and sexes – there is some sense of *community* that unites this group, one that comes from shared experiences, and a largely shared politics (queer people are predominantly left-wing).^42^ This shared politics is no accident; queer people’s experience of oppression can enable a clearer understanding^43^ of the oppression that other marginalized groups face, which lends itself to political solidarity with these groups.^44^ As a consequence, many queer people can go to a queer space – like the LGBTQ+ society at their university – and feel a sense of camaraderie with the other people there. (Though the queer community’s many problems should not be minimized; in many queer spaces, racism, biphobia, and transphobia are still rife.)

But as more and more previously-straight, cisgender people took AET, and became new members of the queer community, the bonds that unite queer people would weaken. Proportionally fewer queer people would have experienced oppression, and proportionally fewer would have the political sympathies that such experience fosters. Queer people would become more of an *agglomeration* of people who happen to have non-normative sexual orientations, genders, and sexes, and less of a *community*. Eventually, sexual orientation might come to have something like the social salience of hair colour, rather than the crucial significance that it has today.^45^

Some might assume that this “fading out” of the queer community would be a bad thing for queer people. But I think that assumption would be too quick. In fact, some queer theorists have suggested that a world in which sexual orientation categories no longer have social salience is the kind of world that we should aspire to.^46^ Such a world would not be ruled by heteronormativity, and people would feel more sexually free.

### Pan-Normativity?

But perhaps a post sexual orientation utopia would not come to be. As more people took AET, pansexuality might simply become the new ‘straight,’ with heterosexuals joining gays and lesbians as oppressed sexual-orientation minorities. And so AET might just bring about a reshuffling of the sexual-orientation hierarchy, rather than its dissolution.^47^

However, I think that we should expect a “pan-normative” world to be much friendlier to sexual orientation minorities than our heteronormative world is.

Under heteronormativity’s reign, man-woman relationships are celebrated, while other romantic pairings are devalued and discriminated against. But if pansexuality became the norm, romantic pairings between people of the same gender and between people of different genders would both be accepted. So you would not have to fear holding your same-gender lover’s hand while walking in public, or have to keep them a secret from your family to avoid being disowned. Perhaps, in a mostly-pansexual world, gays, lesbians, and heterosexuals would feel some sense of alienation from their pansexual peers. But it is difficult to see how anything close to the levels of sexual orientation oppression that we see today could be sustained in a pan-normative world. A world in which pansexuality is celebrated would be a world in which all sexualities are accepted.

### Unknown Change

Some might express concern not over a specific, feared consequence of AET, but over the fact that we know so little about what the full consequences of AET would be, if it were unleashed into society. Given that these consequences could be vast, and are surrounded by so much uncertainty, perhaps the safest thing to do is to err on caution’s side, and not develop AET at all. (Conservative political theorists often raise this type of argument to oppose radical social change; see Burke 1870.)

I think that there is an element of truth in this argument. We should try to think through the possible ramifications of AET carefully – and this paper is only the starting point.^48^ But if, after giving due consideration to the issue, we cannot think of any negative societal consequences that would rival, in moral significance, the benefits I have articulated here – then we should resist appealing to “unknown risks” to forestall AET’s development indefinitely.

That would be, after all, the same desperate argumentative move that some opponents of gay marriage made,^49^ unable to point to any convincing harms that would come from marriage equality. And it would fail for the same reason here as it did there: given the compelling moral reasons we have to fight discrimination and promote romantic liberty, vague unease about change should not stand in our way.

For too long, nature and society have worked to shape and constrain our romantic desires. But these conspirators – while aesthetically discerning – are morally blind. If we can use technology, one day, to defy their directives, our hearts would be better for it.

## Footnotes

1 Some define “romantic” narrowly, and distinguish between romantic attraction and sexual attraction. However, I use “romantic” in the broadest sense; attraction that is purely sexual would fall under its umbrella.

2 I use “queer” as an umbrella term to refer to sexual-orientation minorities (i.e. non-heterosexuals) and sex or gender minorities (i.e. people who are trans, intersex, or non-binary).

3 On love-enhancing technology, see Savulescu & Sandberg’s seminal 2008 paper; and for the best recent work on the topic see Earp (2019) and Earp and Savulescu (2020).

4 On diminishment, see Earp, Wudarczyk, Sandberg, & Savulescu (2013) and Aurenque and McDougall (2013).

5 The existing literature has focused on future technologies that would target sexual orientation. See Aas and Delmas (2016) on why scientific research into these technologies should be banned, and Southan (2019) for a more optimistic view. It should be noted that these types of technologies are importantly different from AET, since AET aims to expand one’s physical attractions generally, not target sexual orientation specifically.

6 In our current heterosexist world, however, Delemas and Aas argue that orientation-changing technology should not be developed, because of the risks such technology would pose to sexual minorities.

7 See McCabe, Cummins, & Romeo (1996).

8 It should be noted that partnerships between people who are not especially compatible with each other can still produce stable, happy, and loving bonds, as Savulescu and Sandberg (2008) point out in the case of arranged marriages (p. 40).

9 For a thoughtful discussion of this point, see Southan (2019).

10 Ansari and Klinenberg (2015) have raised this worry with respect to the increase in romantic options due to dating apps and the rise of big cities; Julian Savulescu (personal communication, 2019) has raised it as an objection to AET. See also Dworkin (1982) on why having fewer options is sometimes rationally preferable.

11 Brake (2012) defines amatonormativity as “the assumption that a central, exclusive, amorous relationship is normal for humans, in that it is a universally shared goal, and that such a relationship is normative, in that it should be aimed at in preference to other relationship types. Brake argues that amatonormativity is harmful because it “devalues friendships and other caring relationships…and relegates friendships and solitudinousness to cultural invisibility” (p. 89).

12 See Eaton’s (2016) summary of the evidence (p. 9–10)

13 However, excess weight increases the risk of serious illness and death from Covid-19, according to Public Health England (2020)- though see Ward (2020) for a critique.

14 To counter lookism, Minerva (2017) argues that we should not only seek to expand societal beauty standards, but also make cosmetic surgery more accessible for those at the bottom of the attractiveness scale. But as Minerva acknowledges, the former kind of intervention has the weakness of being difficult and slow, and the latter intervention has the disadvantage of reinforcing restrictive beauty standards. AET would be an attractive third option, avoiding the limitations of the other approaches.

15 I define “pansexual” as attracted to people of all genders.

16 Summarizing the scientific literature, Earp and Vierra (2018) explain that “apart from a smattering of non-generalizable, anecdotal reports of dubious validity to the contrary, [sexual orientation change efforts] overwhelmingly do not seem to ‘work.’”

17 Earp and Vierra (2018) explain that “the physical, mental, and other ‘medical’ risks associated with common approaches to [sexual orientation change efforts], though difficult to estimate with any precision, can be extremely severe – up to and including suicide – when they do occur.”

18 I follow the LGBTQ+ rights organization Stonewall (n.d.) in defining sexual orientation in terms of *gender*-attraction as opposed to *sex* attraction, for the purpose of trans inclusivity. In doing so, I heed Sally Haslanger’s call (recently invoked by Dembroff 2016, p. 3 and Srinivasan 2017) to consider not only the descriptive accuracy of our concepts, but also the political and moral purposes that they serve (Haslanger 2000).

19 I leave open the question of how, precisely, to define gender, but a morally acceptable definition must respect the gender identifications of people who are trans, as Jenkins (2016) affirms. For further discussion of the concept of gender, see Salett Andler (2017) and Barnes (2019).

20 I thank the audience at the Society for Applied Philosophy Annual Conference for raising this point.

21 See also Sanchez (2017).

22 The possibility that AET could expand a person’s sexual orientation means that it also comes with unique prudential risks. Straight users, after taking AET, might experience some of the harms of homophobia. And both straight and gay users might expose themselves to the harms of biphobia; people who are bisexual often experience discrimination and erasure not only by heterosexuals, but also within the queer community – and, as a result of this ‘double discrimination,’ experience higher rates of depression and anxiety than gay people, according to some studies (see Ross et al. 2018 for a systematic review). For AET with potentially orientation-expanding effects to be in users’ prudential interests, it might need to be taken at a place and time where homophobia and biphobia was sufficiently low.

23 Both of these non-harm-based concerns were raised by attendees of a queer seminar, where I presented an early draft of this paper.

24 For example, opponents of gay rights sometimes claim that children raised by same-sex parents fare worse than children raised by a mother and father, though this claim is not supported by the balance of evidence (Power and Crouch 2017).

25 Talk show host Michael Strahan (2019) recently invoked this principle in support of gay marriage, underscoring its persuasiveness and common-sense appeal: “You’re not hurting me, you’re not hurting anyone else, go ahead and do your thing. Plain and simple.”

26 See Feinberg (1990) for an overview of possible harmless wrongs.

27 For instance, one can, contra consequentialism, accept deontological constraints against harming, while also maintaining that harmless acts cannot be morally wrong.

28 For instance, harms can be non-experiential as well as experiential, under some accounts (Spena 2010).

29 Of course, harm cannot be *too* widely construed or the first clause of the response – that queer people are not harming anyone by being queer – could be threatened. For accounts of harm that are both wide enough to accommodate non-experiential harms, and narrow enough to exclude the distress that homophobes might experience at the sight of queer people (and the frustration of their other-regarding preferences), see Mill (1859) Chapter IV, par. 3 and Feinberg (1984), Chapter 2.

30 To accommodate non-identity cases, a more precise statement of the principle would be, “An action cannot be morally wrong if it does not harm anyone or reduce aggregate wellbeing.”

31 It should be noted that not all profferings of harmless wrongs can be explained by bigotry (e.g. harmless trespass).

32 Earp (2016) and Earp and Vierra (2018) have also pointed out that future orientation-changing technology could undermine the “it’s not a choice” justification for queer rights, and have emphasized the importance of appealing to a stronger, non-technologically-contingent justification.

33 See also Weber (2012) on the erasure of both of these groups.

34 See also Vierra (2016) on bad-faith uses of a “gay-for-a-day pill.”

35 On the value of casual sex, Settegast (2018) writes: “Connecting sexually with a stranger sometimes indeed may be easier than with a long-term partner, for long-standing relationships can be fraught with difficulties that make it hard to be sexually open and uninhibited. Casual sex involves no need to consider the history or future of a relationship but places partners in a situation that is purely sexual, unburdened by anything beyond their joint pursuit of enjoyment, and thereby sometimes more readily enables a spontaneous and uncomplicated, even if transitory sense of connection. Provided the chemistry is right, successfully engaging in a ‘lovely fling’ may, on occasion, even result in feelings of general benevolence – that such an intimate, positive experience of affirmation and community is in fact possible with otherwise strangers.”

36 I will revisit this issue in Part IV, and consider what it would mean for the queer community if a very large proportion of its members lack these experiences and struggles.

37 Rini (2018) defines a “microaggression” as “a small insulting act made disproportionately harmful by its part in an oppressive pattern of similar insults.”

38 It seems unlikely that there would be a deontological constraint against taking AET (on the grounds that taking AET increases the likelihood that a person will make insensitive comments in queer spaces). Deontological constraints are most plausible when it comes to harms that are: (1) severe (2) certain to occur, and (3) intentionally inflicted. But AET use has none of these features. (It is not certain that a previously-straight AET user will make any insensitive comments at all, and – because I have stipulated that they are acting in good faith – any insensitive comments that they did make would not be intentional.)

39 In addition to supporting the moral permissibility of orientation-expanding AET, such a prerogative can also bolster the case for queer rights discussed earlier in Part III; in addition to not harming anyone by being queer, queer people have a positive right to live their romantic lives as they choose.

40 See Frederick and Loewenstein (1999).

41 Supra note 7.

42 Though see Fischel (2020), who critiques the idea of the queer community as a unified group, highlighting the vastly different treatment at the hands of the police of trans women of color versus white gays.

43 Feminist standpoint theorists have pointed out the epistemic privileges that the experience of oppression confers.

44 O.n queer solidarity, see LGSMigrants (2020).

45 The fading out of the queer community need not mean a disappearance of the type of left-wing *politics* that the queer community is associated with; political activism can be organized along affinity-based rather than identitarian lines. For a defence of affinity-based politics over identity politics, see Haraway (1985).

46 Foucault (1976) argues that, while people have engaged in homosexual sex throughout history, the nineteenth century invention of the homosexual as an identity category, or “species” (p. 43), enabled sexuality to be more heavily controlled. Similarly, Butler (1993) writes that “identity categories [like lesbian and gay] tend to be instruments of regulatory regimes” (p. 308). More recently, Dembroff (2016) has proposed a new taxonomical schema of sexual orientation that eliminates the categories of heterosexual and homosexual, arguing that these categories reinforce heteronormativity (p. 19).

47 I thank an anonymous reviewer for raising this point.

48 One important question is whether research into AET would create a ‘dual use risk’ – where findings might be misapplied by bad actors in order to create high-tech gay ‘conversion therapies.’ Such ‘therapies’ (as I discussed in Part III) would threaten to decimate queer populations. This is an important concern that I leave to future work (and those with the relevant scientific and regulatory expertise) to explore in the depth that it deserves. However, several preliminary considerations suggest that it does not weigh against AET’s development decisively. First, the development of AET might not involve conducting any research into sexual orientation at all. For example, attraction-expansion might be observed as a side effect of a drug with some other use (just as lowered libido is a side effect of some anti-depressants), and then that drug might be used by people ‘off-label,’ as AET. Second, AET might be developed in a context in which high-tech conversion therapies had already been developed by bad actors. Third, dual use risk could be minimized if conversion therapy is banned (as it currently is in some countries), and if researchers refrain from publishing research that might be misapplied in this way. Fourth, if AET’s adoption helps bring about a society that is more accepting of queer rights (as I have argued it might in this section), then this would reduce pressure on queer people to undergo (low and high-tech) conversion therapies.

49 See e.g. Carpenter’s (2009) summary of the traditionalist case against marriage equality (which Carpenter rejects) (p. 94). See also Wax (2005).
